# The Efficacy and Safety of Esmolol for Septic Shock: A Systematic Review and Meta-analysis of Randomized Controlled Trials

**DOI:** 10.3389/fphar.2021.682232

**Published:** 2021-06-01

**Authors:** Po Huang, Xiangchun Zheng, Zhi Liu, Xiaolei Fang

**Affiliations:** Beijing Dongfang Hospital, Beijing University of Traditional Chinese Medicine, Beijing, China

**Keywords:** septic shock, tachycardia, esmolol, meta-analysis, systematic review

## Abstract

**Purpose:** The meta-analysis aims to identify whether septic shock patients can benefit from esmolol.

**Materials and Methods:** The relevant studies from MEDLINE, Cochrane Library, Embase were searched by two independent investigators using a variety of keywords. Stata software (version 12.0, Stata Corp LP, College Station, TX, United States)was used for statistical analysis.

**Results:** A total of 14 studies were identified and incorporated into the meta-analysis. For overall analysis, the treatment of esmolol was associated with decreased 28-day mortality (RR = 0.66, 95% CI = 0.56–0.77, *p* < 0.001). Meanwhile, our analysis found that, esmolol could decrease HR (SMD: −1.70; 95% CI: [−2.24−(−1.17)], cTnI (SMD: −1.61; 95% CI: [−2.06−(−1.16)] compared with standard treatment. No significant differences between the two groups were found in MAP, Lac, CI, and SVI.

**Conclusion:** The findings of this meta-analysis intend to demonstrate that septic shock patients with high heart beats rate might be benefit from esmolol treatment despite enough fluid resuscitation. While, dependent on the study published, with the further development of septic shock, the positive impact of esmolol varies. The appropriate heart rate change interval cannot be confirmed, further high-quality and large-scale RCTs should be performed to verify it and screening more suitable heart rate levels.

**Systematic Review Registration:** CRD42021239513

## Introduction

Septic shock is typically defined in a clinical setting by low systolic pressure (≤90 mmHg) or mean arterial blood pressure (≤65 mmHg) accompanied by signs of hypoperfusion (Berger Rebecca, 2017). For septic shock, vasoactive drugs are important means to maintain the stability of hemodynamics and ensure the perfusion of major organs (Seymour Christopher and Rosengart Matthew, 2015). Norepinephrine (NE) is the first-line drug for septic shock (Jean-Louis and Post Emiel, 2016). While, long term application of norepinephrine can lead to adverse reactions, such as arrhythmia, the most common of which is tachycardia (Schwinger Robert, 2021). Ventricular tachycardia is a direct response to catecholamine exposure and an independent prognostic factor in patients with septic shock (Hasegawa, et al., 2021).

As a common drug of cardiovascular disease, β-receptor blocker has the function of controlling heart rate, anti-arrhythmia, improving myocardial remodeling, metabolism and immune regulation (Szentmiklosi, et al., 2015). Esmolol is a highly selective β1 receptor blocker with short half-life, which can be pumped drip. It has the advantages of rapid onset, good tolerance and easy regulation, which is the most commonly used preparation in critical care medicine (Alberto, et al., 2021). Considering the negative inotropic effect of β-receptor, it can reduce myocardial contractility and cardiac output, which has the potential risk of further aggravating shock. Thus, β-receptor has not been recommended in the guideline (Shankar-Hari, et al., 2016).

Recently, there have been a number of randomized controlled trials comparing esmolol with standard treatment for septic shock. However, there is still much controversy in the influence of esmolol on mortality, MAP, CI and HR. In view of this, it is necessary to conduct a systematic review to evaluate the efficacy and safety of esmolol in the treatment of septic shock, so as to provide evidence-based evidence for the treatment of septic shock.

## Methods

This meta-analysis was conducted according to the recommendations and checklist from the preferred reporting items for systematic review and meta-analysis (PRISMA) statement (Panic et al., 2013).

### Search Strategy

We searched the relevant studies from Pubmed, Cochrane Library, Embase from their inception to March 2021, conference proceedings, and reference lists of relevant articles were also searched.

### Eligibility Criteria of Original Studies

Inclusion criteria: 1) Participants: we included septic shock patients with tachycardia, regardless of the gender and ethnicity; 2) Interventions: Esmolol; 3) Control: Standard therapy; 4) Outcomes: Primary outcomes: 28-day mortality; secondary outcomes:heart rate (HR), mean arterial pressure (MAP), lactate level, cardiac index (CI), stroke volume index (SVI), cardiac troponin I (cTnI), adverse events; 5) The study design was randomized controlled trial (RCT).

Exclusion criteria: a study with duplicate publication and/or abstract only.

### Study Selection

Two reviewers independently identified studies through inclusion criteria by screening the title and abstract of each record and retrieved their full-text if necessary. Any disagreement between the two reviewers was solved with a discussion with a third reviewer. Otherwise, the agreement was accomplished by a consensus.

### Data Extraction and Quality Assessment

We designed a pre-defined data extraction form and two reviewers independently extracted the following information from the selected trials: the first author, published year, sample size, intervention, control and outcomes. Any disagreement between two reviewers was discussed with the third reviewer until a consensus was reached.

Quality assessment was performed by two reviewers. RCTs were scored as per the Jadad Scale. According to its principle, one to three indicating a low-quality study and four to five indicating a high-quality study, the maximum of Jadad score is five.

### Data Synthesis

Stata software (version 12.0, Stata Corp LP, College Station, TX, United States) was used for statistical analysis. According to the Cochrane Handbook of Systematic Reviews, we chose risk ratios (RRs) and 95% confidence intervals (CIs) as the appropriate parameters to evaluate the dichotomous outcomes. In terms of continuous outcomes, the mean difference and its 95% CI were used. Between-study heterogeneity was evaluated using an I^2^ test (25% or lower is defined as low heterogeneity, 50% as moderate heterogeneity, 75% as high heterogeneity). The fixed-effect model was applied if there was no or low heterogeneity, and pooled RRs were estimated using the Mantel-Haenszel method. Publication bias was assessed if there are more than ten studies in one outcome. All hypotheses were tested at the alpha = 0.05 level.

## Results

### Description of Included Studies

**FIGURE 1:**
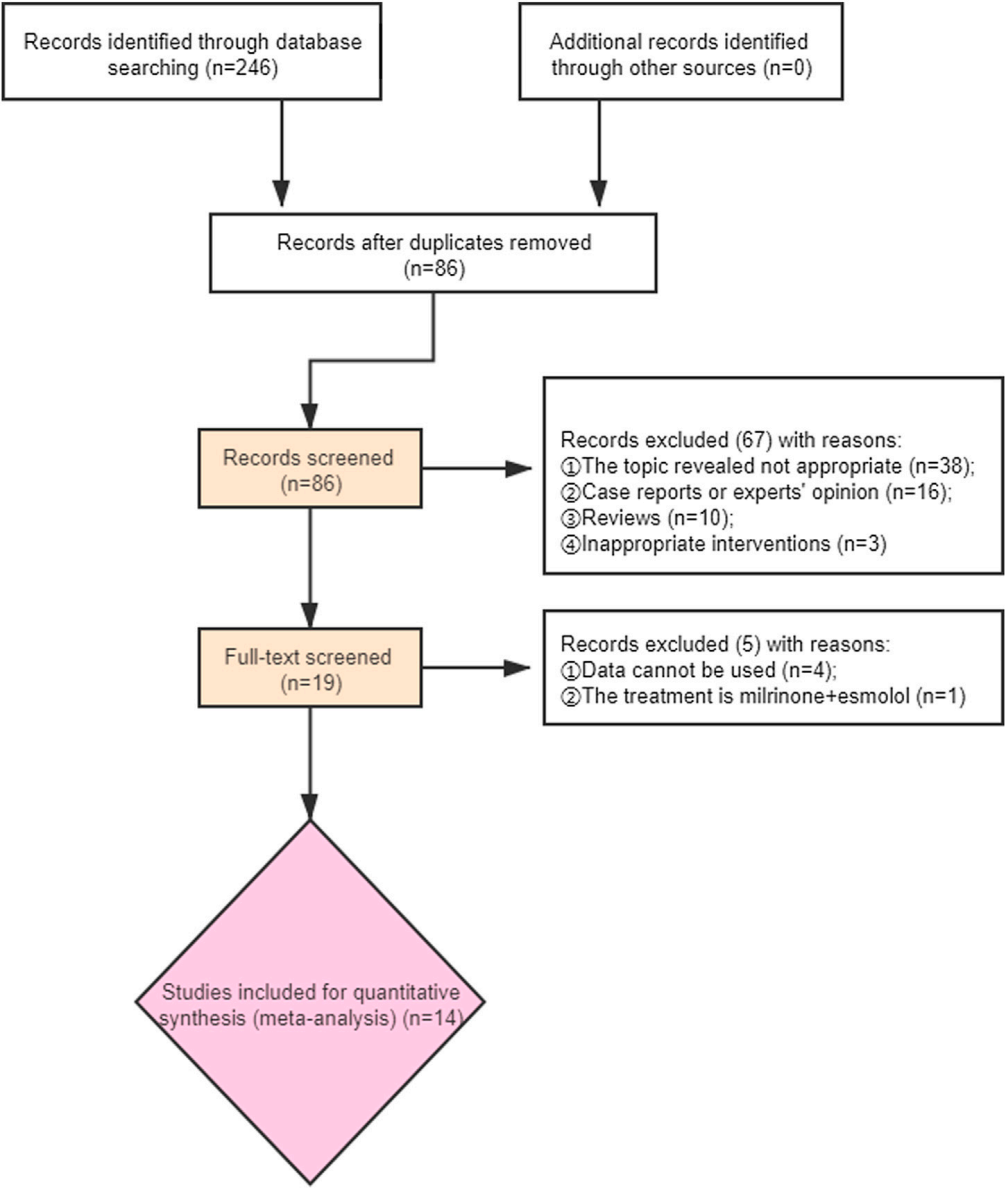
Flow chart of included studies selection.

We identified 246 records based on this search strategy, and 86 potentially eligible records were obtained after removing duplicate publications. After screening the titles and abstracts, a total of 67 studies were excluded. Fourteen studies with 927 participants were included (Figure 1). The characteristics of the included studies are presented in Table 1. Risk of bias for the included studies were assessed by the Jadad scale, and the result are presented in Supplementary Table S1.

**TABLE 1:** the characteristics of the included studies.

Study	No. of participants	Intervention		Outcomes
		Experimental group	Control group	
Morelli et al. (2013)	<I>N</I> = 154 (T = 77; C = 77)	Esmolol	ST	28-day mortality, HR, MAP
Yang S et al. (2014)	<I>N</I> = 41 (T = 21; C = 20)	Esmolol	ST	MAP, HR, Lac, CI, SVI, cTnI
Liu et al. (2015)	<I>N</I> = 48 (T = 24; C = 24)	Esmolol	ST	28-day mortality, HR, MAP, Lac
Wang et al. (2015)	<I>N</I> = 60 (T = 30; C = 30)	Esmolol	ST	28-day mortality, HR, MAP, Lac, CI, SVI, cTnI, adverse events
Wang et al. (2017)	<I>N</I> = 60 (T = 30; C = 30)	Esmolol	ST	28-day mortality, HR, MAP, Lac, CI, SVI
Chu and Chen, (2017)	<I>N</I> = 70 (T = 35; C = 35)	Esmolol	ST	28-day mortality, HR, MAP, Lac, CI
Wang et al. (2017)	<I>N</I> = 38 (T = 19; C = 19)	Esmolol	ST	28-day mortality, HR
Li. (2017)	<I>N</I> = 70 (T = 35; C = 35)	Esmolol	ST	HR, MAP, Lac, CI, SVI, cTnI, adverse events
Wang et al. (2018)	<I>N</I> = 50 (T = 25; C = 25)	Esmolol	ST	Lac, CI, cTnI
Tao. (2018)	<I>N</I> = 64 (T = 32; C = 32)	Esmolol	ST	HR, MAP, Lac, CI, SVI
Liu et al. (2019)	<I>N</I> = 100 (T = 50; C = 50)	Esmolol	ST	28-day mortality, HR
Yang C et al. (2019)	<I>N</I> = 44 (T = 22; C = 22)	Esmolol	ST	28-day mortality, HR, MAP, Lac, CI, SVI, cTnI
Zhou et al. (2019)	<I>N</I> = 64 (T = 32; C = 32)	Esmolol	ST	28-day mortality
Zhou et al. (2019)	<I>N</I> = 64 (T = 32; C = 32)	Esmolol	ST	cTnI, adverse events

### Primary Outcome

**FIGURE 2:**
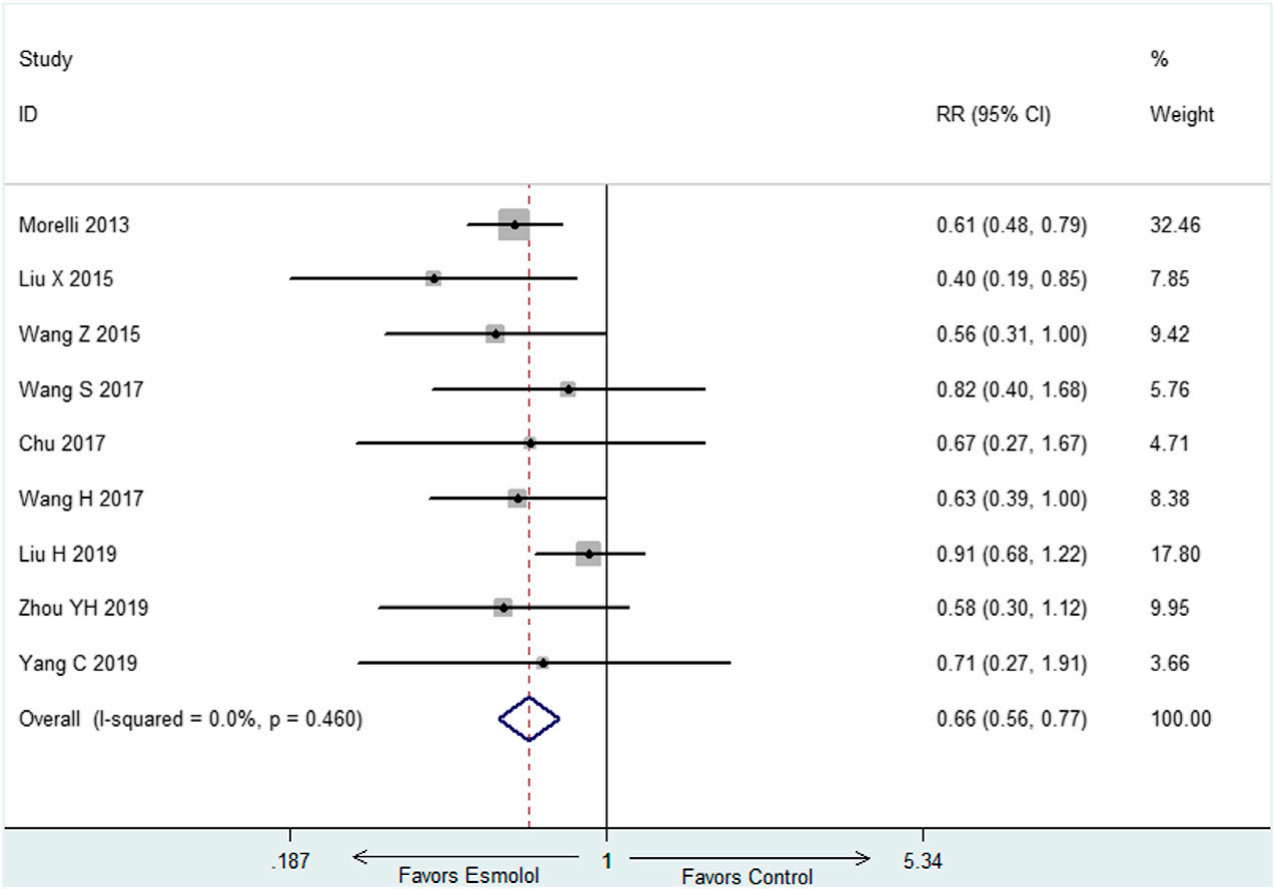
Forest plot of 28-day mortality.

The primary outcome is 28-day mortality. Nine studies (Morelli et al., 2013; Liu et al., 2015; Wang et al., 2015; Wang et al., 2017; Chu and Chen, 2017; Wang et al., 2017; Liu et al., 2019; Yang C et al., 2019; Zhou et al., 2019) with 708 participants reported 28-day mortality. The result showed that 28-day mortality is lower in esmolol group than control group (RR = 0.66, 95% CI = (0.56,0.77), *p <* 0.01) (Figure 2).

### Secondary Outcomes

#### Heart Rate at 72-h

**FIGURE 3:**
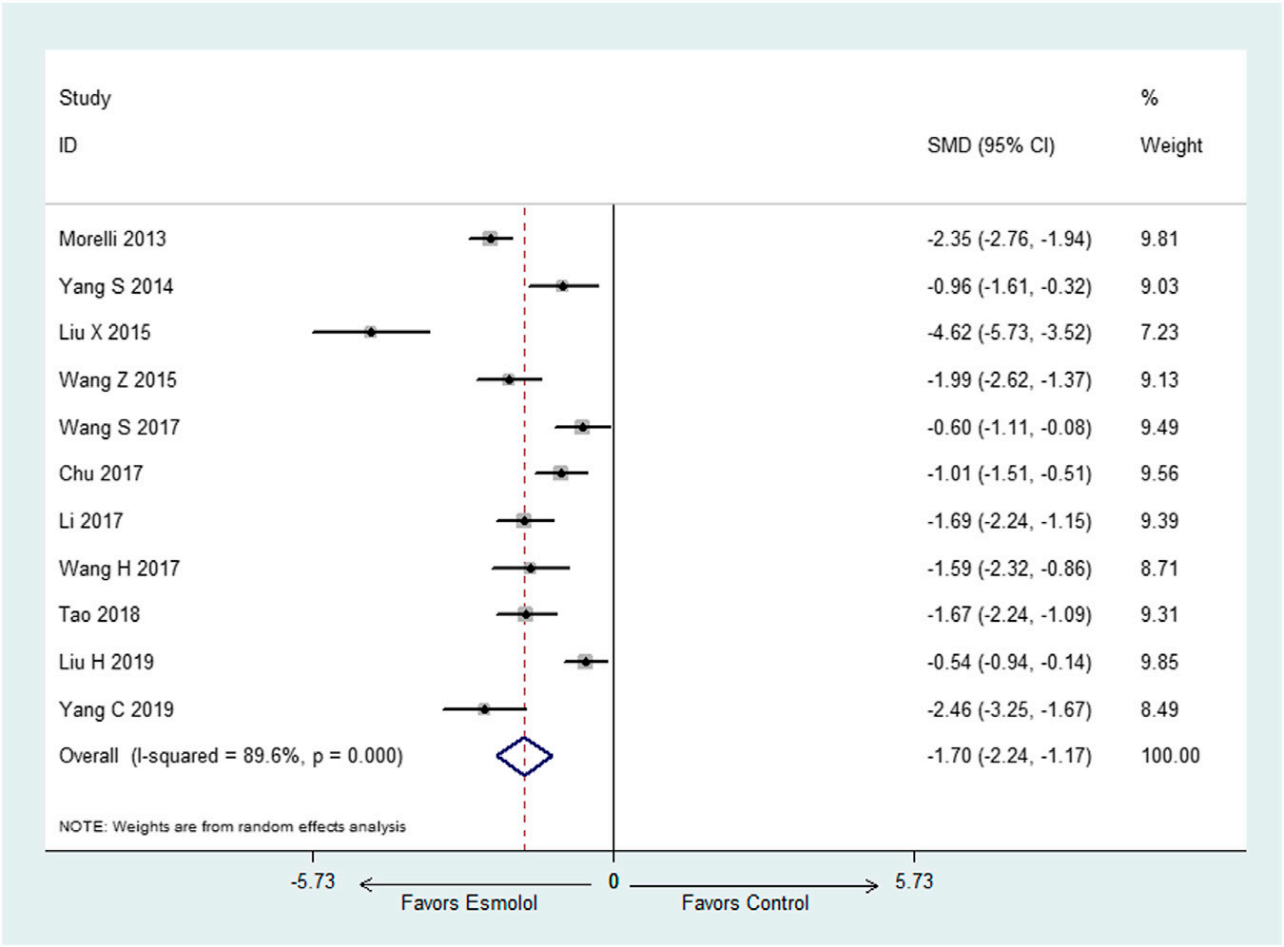
Forest plot of HR.

Eleven studies (Morelli et al., 2013; Yang et al., 2014; Liu et al., 2015; Wang et al., 2015; Wang et al., 2017; Chu and Chen, 2017; Wang et al., 2017; Li, 2017; Tao, 2018; Liu H et al., 2019; Yang C et al., 2019) employed HR at 72-h as outcome measure. There was obvious heterogeneity among these included studies, thus, random effect model was utilized for statistical analysis. The results indicated that esmolol plus standard treatment could decrease HR when compared with standard therapy (SMD: −1.70; 95% CI: [−2.24−(−1.17)] (Figure 3).

### Mean Arterial Pressure at 72-h

**FIGURE 4:**
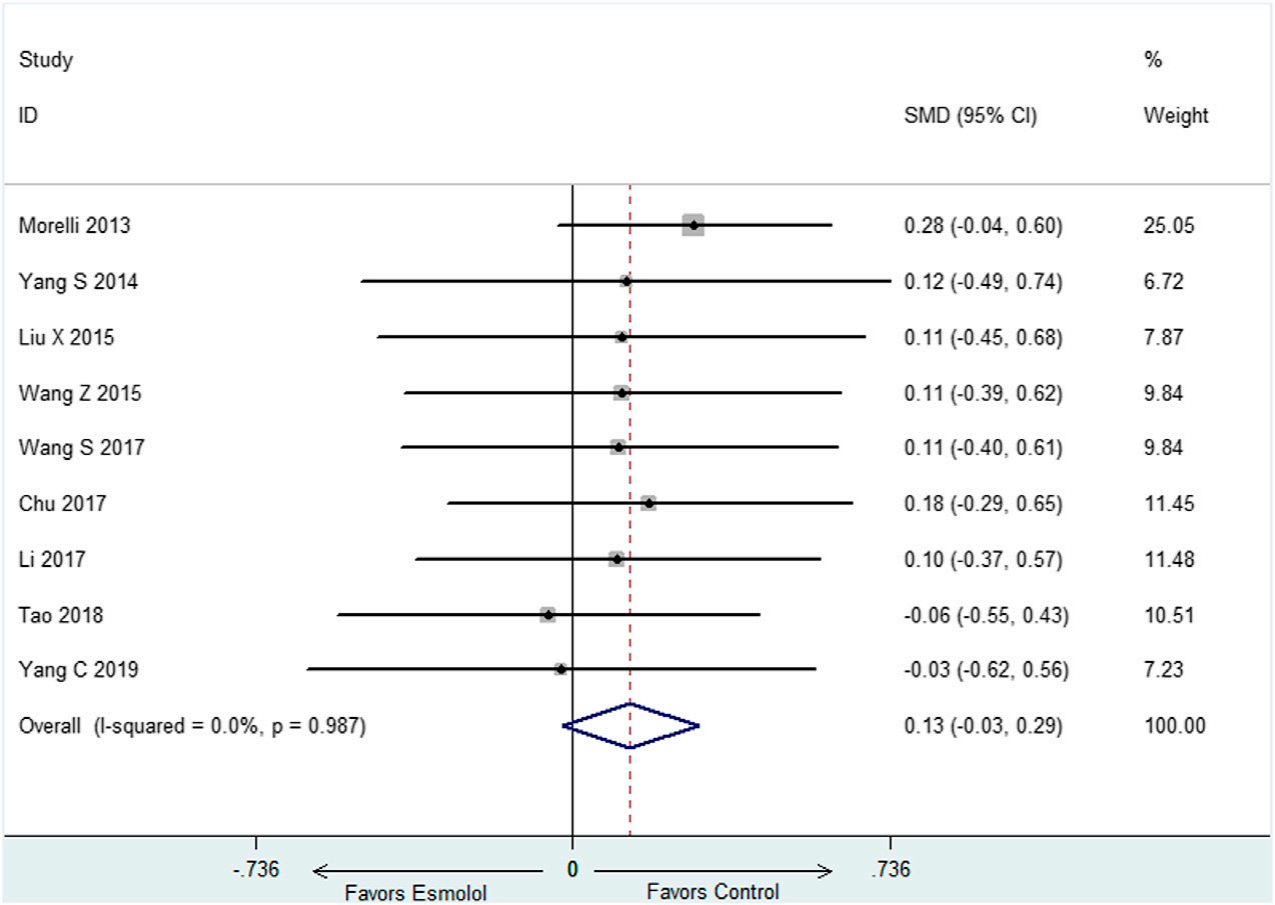
Forest plot of MAP.

There were nine studies (Morelli et al., 2013; Yang et al., 2014; Liu et al., 2015; Wang et al., 2015; Wang et al., 2017; Chu and Chen, 2017; Li, 2017; Tao, 2018; Yang C et al., 2019) reported MAP at 72-h, we selected fixed effect model since there was no heterogeneity in both of the two subgroups (I^2^ = 0). The meta-analysis showed that compared with standard therapy, adding esmolol has no influence on MAP at 72-h (SMD: 0.13; 95% CI: −0.03–0.29) (Figure 4).

### Cardiac Troponin I at 72-h

**FIGURE 5:**
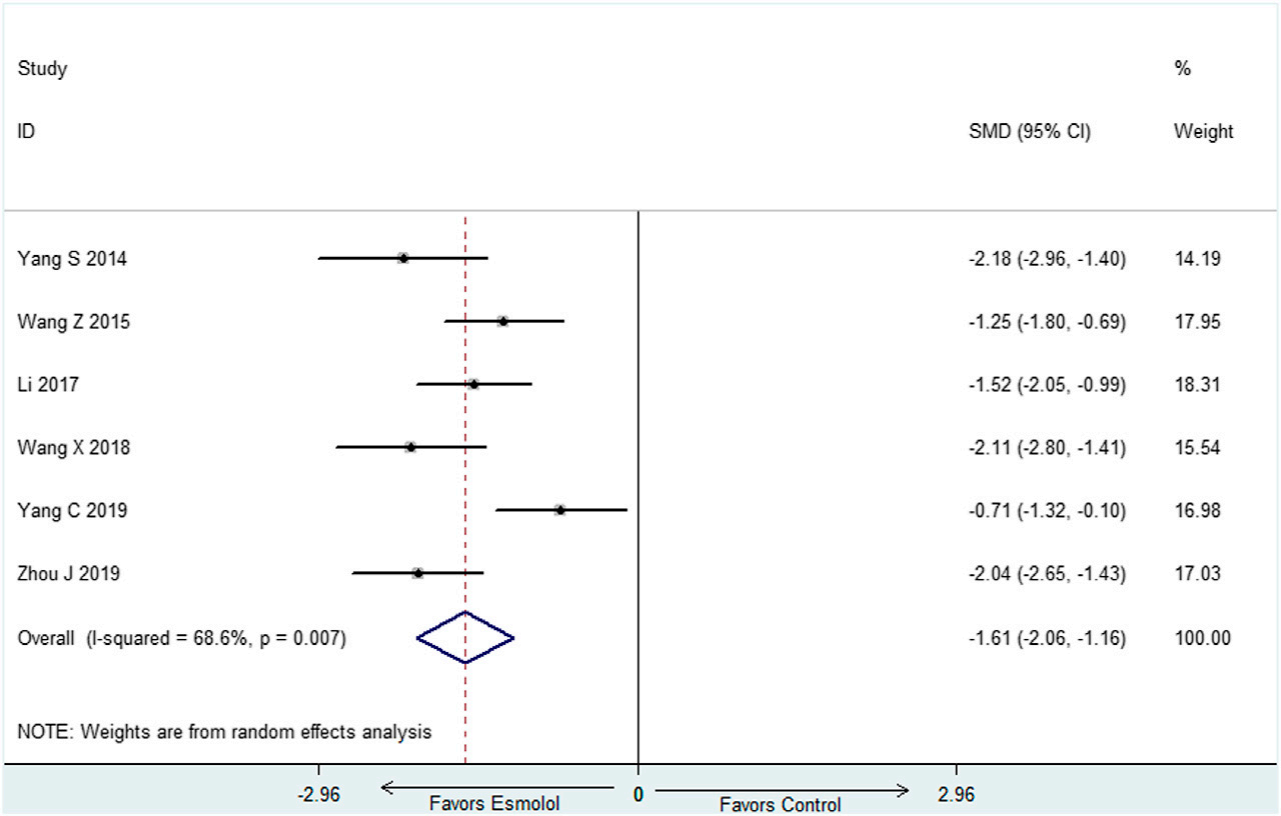
Forest plot of cTnI.

Six studies (Yang et al., 2014; Wang et al., 2015; Li, 2017; Wang et al., 2018; Yang C et al., 2019; Zhou et al., 2019) with 329 participants reported cTnI at 72-h, random effect model was utilized (I^2^ = 68.6%). The result demonstrated that cTnI at 72-h was decreased in the group of esmolol compared with standard treatment (SMD: −1.61; 95% CI: [−2.06−(−1.16)] (Figure 5).

### Lactate Level, Cardiac Index and Stroke Volume Index at 72-h

Random effect models were utilized (I^2^> 75%) in the above three outcomes. The results showed that there was no influence on Lac, CI and SVI at 72-h in the group of esmolol compared with standard treatment (Supplementary Figures S1–S3).

### Adverse Events

Three studies reported adverse events and there was no significant difference in the adverse effects among the two groups.

## Discussion

### Summary of Findings

The analysis aims to identify the efficacy and safety of esmolol for septic shock. The results showed a survival benefit with esmolol compared with standard treatment. Meanwhile, our analysis found that the use of esmolol could decrease HR and cTnI with no influence on MAP, CI, SVI, and Lac (Table 2). Thus, based on the results of our analysis, esmolol is recommended for septic shock patients with tachycardia.

**TABLE 2:** Summary of meta-analysis.

Outcomes	No. of studies	No. of participants	Effect size (95% CI)	I^2^
28-day mortality	9	708	RR, 0.66 (0.56, 0.77)	0
HR	11	749	SMD, −1.70 (−2.24, −1.17)	89.6%
MAP	9	611	SMD, 0.13 (−0.03, 0.29)	0
cTnI	6	329	SMD, −1.61 (−2.06, −1.16)	68.6%
Lac	9	507	SMD, −0.16 (−0.80, 0.49)	91.7%
CI	7	399	SMD, 0.13 (−0.82, 1.08)	94.9%
SVI	6	339	SMD, 0.67 (−0.04, 1.39)	89.8%

HR, heart rate; MAP, mean arterial pressure; Lac, lactate; CI, cardiac index; SVI, stroke volume index; RR, relative risks; WMD, weighted mean difference.

### Why, When and in Whom Esmolol Are Beneficial?

As we all known, Morelli et al. (Morelli et al., 2013) conducted the first randomized controlled study to evaluate the efficacy and safety of esmolol in septic shock. Surprisingly, this study reported a substantial reduction in mortality with a 28 days mortality of 49.4% in the esmolol group and 80.5% in the control group. Meanwhile, it is interesting that norepinephrine and resuscitative fluid volume requirements were reduced in the esmolol group whilst maintaining MAP ≥65 mmHg. This study has raised questions and stimulated interest into why, when and in whom esmolol are beneficial.

First, septic shock related cardiac dysfunction is mainly associated with prolonged catecholamine exposure and β receptor overactivation (Suzuki et al., 2017). Long-term excessive stimulation can lead to imbalance of myocardial energy supply. Rapid ventricular rate is a direct response to catecholamine exposure, and it is also an independent prognostic factor. The recent study demonstrated that the application of esmolol can up-regulate the β-receptor on the surface of myocardial cells, restore heart rate variability and sensitivity to catecholamine (Orbegozo et al., 2014). In addition, the negative frequency effect of β-blocker prolonged left ventricular diastolic period, increased left ventricular ejection fraction and left ventricular blood volume (Harada et al., 2020). What’s more, the related studies reported that esmolol can reduce the expression of chemokines and inflammatory factors in cardiac dysfunction (Karlsen et al., 2019).

Second, the appropriate timing of esmolol administration has not yet been determined. Tachycardia is the main compensatory mechanism to prevent the decrease of cardiac output (Mohammed et al., 2021). At this time, if the heart rate is reduced blindly, it may lead to organ perfusion insufficiency and damage organ function. Sufficient fluid resuscitation is important for septic shock, whereas, esmolol maybe useful if the heart rate rise above 100 despite initial resuscitation. Usually, if tachycardia still exists after sufficient fluid resuscitation and exclusion of common causes such as hypovolemia, it is considered that it may be caused by sympathetic excitation, and esmolol is more appropriate at this time. However, the standard of sufficient fluid resuscitation is still lack, which could be evaluated by bedside echocardiography and Pulse Contour Cardiac Output (PiCCO).

Last but not least, relevant studies have shown that the increase of heart rate is positively correlated with the mortality of patients with septic shock, so septic shock with tachycardia is the indication of esmolol (Lee Young et al., 2019; DellaVolpe Jeffrey et al., 2015; Emmanuel et al., 2018). Most of the included studies selected septic shock patients with HR ≥ 100 beats/min. However, the range of heart rate is too large, and the upper limit of heart rate is not limited. Liu et al. reported that the 28-day mortality was higher in esmolol group compared with control group in septic shock patients with HR ≥ 120, though there was no statistical difference between two group (Liu et al., 2019). This finding suggests that patients with septic shock whose heart rate is more than 120 beats/min may not benefit from esmolol. After further review of the included studies, we found that only one study (Liu et al., 2019) performed the subgroup analyze for heart rate in the outcome of mortality. The data demonstrated that there was a positive correlation between heart rate and mortality in both groups. By logistic regression analysis, it was found that 28-day mortality risk of septic shock patients increased by 1.568 times for every 10 beats/min increase in initial heart rate, and increased by 2.207 times for every 10 beats/min increase in overall heart rate after treatment (Liu et al., 2019). Compared with control group, septic shock patients could benefit from esmolol when heart rate rang from 110 to120. Whereas, the benefit cannot be obtained when heart rate rang from 100 to 110 or 120 to 130. Dependent on the study published, with the further development of septic shock, the positive impact of esmolol varies. Based on the existing evidence, the upper limit of heart rate cannot be confirmed, and more studies are needed to confirm the precise heart rate range. Besides, the severity of the disease (such as SOFA and APACHE Ⅱ score), complications, basic heart conditions will affect the efficacy of esmolol. Thus, how to distinguish the benefit patients of esmolol in patients with septic shock needs further study.

### Strengths and Limitations

Though there exit three similar reviews (Yu et al., 2016; Liu et al., 2018; Li et al., 2020), there are some differences between our review and the three reviews. First, we found that some of the included studies in the previous reviews were repetitive studies. Second, most of the studies were not included, which limits the credibility of the results. Third, our review included the largest number of studies and the most comprehensive evaluation index to evaluated the efficacy and safety of esmolol for septic shock. Through our review, we found that through esmolol could decrease 28-day mortality in septic shock, the appropriate heart rate range is unclear, which limited the application in clinic. Through further reading of the included studies, we found that most of the studies set the heart rate interval above 100 beats/min, and they did not set the upper limit. One of the studies found that esmolol had no significant protective effect on death when the heart rate exceeded 120 beats per min (Liu H et al., 2019). Thus, we speculate that esmolol may be more suitable for septic shock patients with heart rate of 100–120 beats/min. Of course, this conjecture needs to be confirmed by more high-quality randomized controlled trials in the future.

Several limitations exit in this review. First, most of the included studies were performed in China, which cannot represent patients with septic shock in other regions. Second, most of the included studies were published in Chinese, only three studies (Morelli et al., 2013; Liu et al., 2015; Wang et al., 2015) were published in English. Third, Since the treatment of septic shock is comprehensive, the effects of other united medication can't exclude.

## Conclusion

The findings of this meta-analysis intend to demonstrate that septic shock patients with high heart beats rate might be benefit from esmolol treatment despite enough fluid resuscitation. While, dependent on the study published, with the further development of septic shock, the positive impact of esmolol varies. The appropriate heart rate change interval cannot be confirmed, further high-quality and large-scale RCTs should be performed to verify it and screening more suitable heart rate levels.

## Data Availability

The original contributions presented in the study are included in the article/Supplementary Material, further inquiries can be directed to the corresponding authors.
