# Supplementary material for: The Efficacy and Safety of Esmolol for Septic Shock: A Systematic Review and Meta-analysis of Randomized Controlled Trials
Source: Front Pharmacol. 2021 Jun 1;12:682232. doi: 10.3389/fphar.2021.682232 (PMC8204042; doi:10.3389/fphar.2021.682232)
Supplement: Supplementary file 1 [file Table1.DOCX]

**Table S1 The quality of included studies**

| Study | The generation of random sequences | Allocation concealment | Blinding method | Reasons for withdrawal and dropout | Total scores |
| --- | --- | --- | --- | --- | --- |
| Morelli A et al, 2013 | 2 | 1 | 0 | 1 | 4 |
| Yang S et al, 2014 | 2 | 1 | 0 | 1 | 4 |
| Liu X et al, 2015 | 2 | 1 | 0 | 1 | 4 |
| Wang Z et al, 2015 | 2 | 1 | 0 | 1 | 4 |
| Wang S et al, 2017 | 2 | 1 | 0 | 1 | 4 |
| Chu et al, 2017 | 2 | 0 | 0 | 1 | 3 |
| Wang H et al, 2017 | 2 | 1 | 0 | 1 | 4 |
| Li et al, 2017 | 2 | 0 | 0 | 1 | 3 |
| Wang X et al, 2018 | 2 | 1 | 0 | 1 | 4 |
| Tao et al, 2018 | 2 | 0 | 0 | 1 | 3 |
| Liu H et al. 2019 | 2 | 1 | 0 | 1 | 4 |
| Yang C et al, 2019 | 2 | 1 | 0 | 1 | 4 |
| Zhou YH et al, 2019 | 2 | 1 | 0 | 1 | 4 |
| Zhou J et al, 2019 | 2 | 0 | 0 | 1 | 3 |
