# Supplementary figures and images for: The Efficacy and Safety of Esmolol for Septic Shock: A Systematic Review and Meta-analysis of Randomized Controlled Trials
Source: Front Pharmacol. 2021 Jun 1;12:682232. doi: 10.3389/fphar.2021.682232 (PMC8204042; doi:10.3389/fphar.2021.682232)

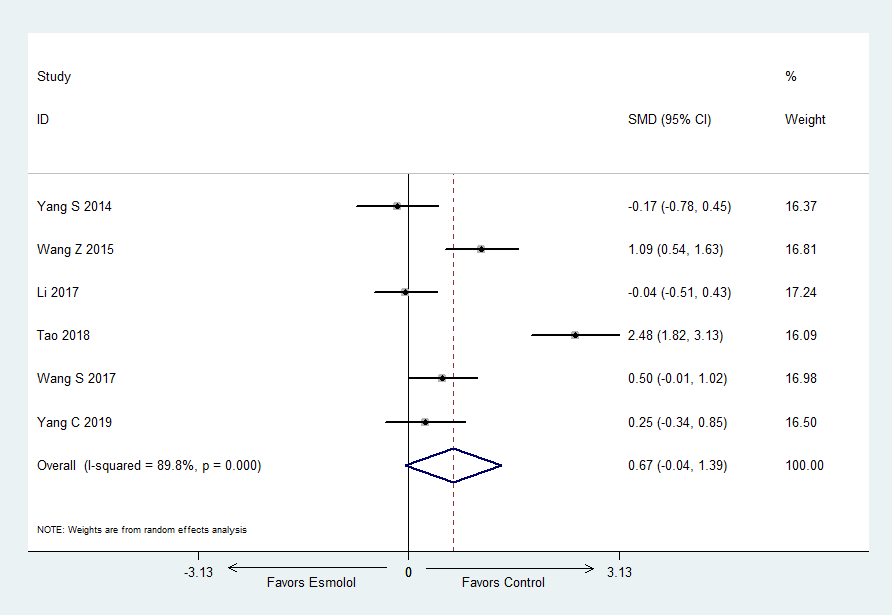

Supplement: Supplementary file 2 [file Image3.TIF]

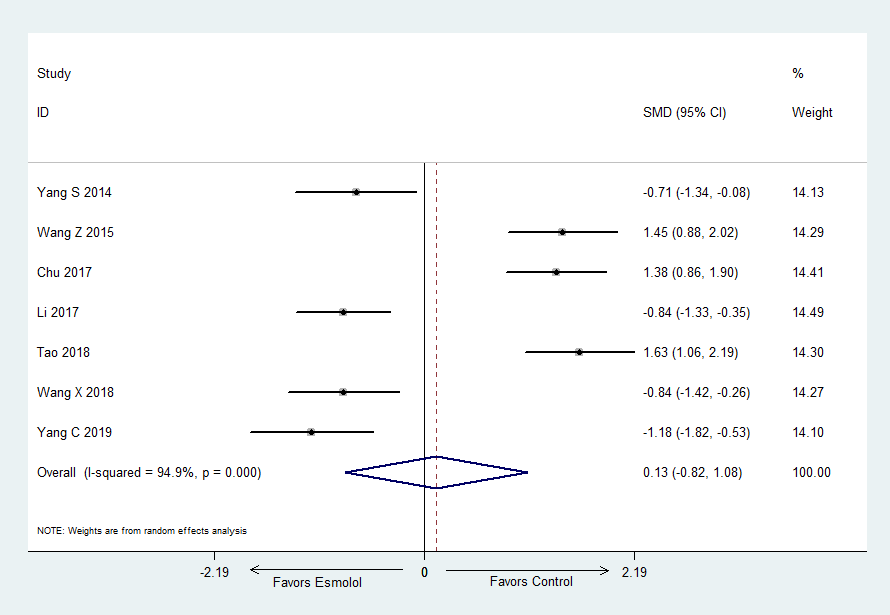

Supplement: Supplementary file 3 [file Image2.TIF]

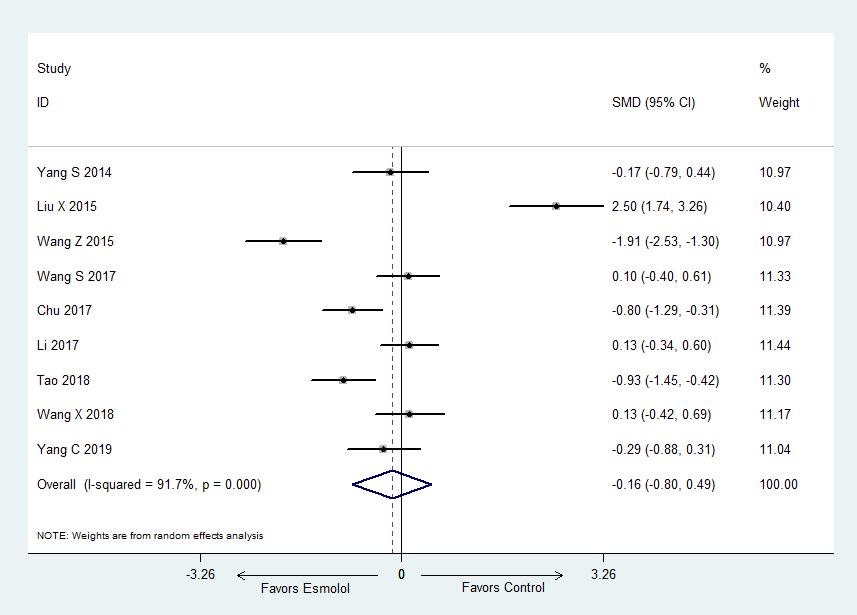

Supplement: Supplementary file 4 [file Image1.TIF]
